# Myostatin Is Elevated in Congenital Heart Disease and After Mechanical Unloading

**DOI:** 10.1371/journal.pone.0023818

**Published:** 2011-09-13

**Authors:** Lawrence T. Bish, Isaac George, Simon Maybaum, Jonathan Yang, Jonathan M. Chen, H. Lee Sweeney

**Affiliations:** 1 Department of Physiology, University of Pennsylvania School of Medicine, Philadelphia, Pennsylvania, United States of America; 2 Department of Surgery, Division of Cardiothoracic Surgery, College of Physicians and Surgeons, Columbia University, New York, New York, United States of America; 3 Department of Medicine, Division of Cardiology, Albert Einstein College of Medicine, New York, New York, United States of America; Medical College of Georgia, United States of America

## Abstract

**Background:**

Myostatin is a negative regulator of skeletal muscle mass whose activity is upregulated in adult heart failure (HF); however, its role in congenital heart disease (CHD) is unknown.

**Methods:**

We studied myostatin and IGF-1 expression via Western blot in cardiac tissue at varying degrees of myocardial dysfunction and after biventricular support in CHD by collecting myocardial biopsies from four patient cohorts: A) adult subjects with no known cardiopulmonary disease (left ventricle, LV), (Adult Normal), (n = 5); B) pediatric subjects undergoing congenital cardiac surgery with normal RV size and function (right ventricular outflow tract, RVOT), (n = 3); C) pediatric subjects with worsening but hemodynamically stable LV failure [LV and right ventricle (LV, RV,)] with biopsy collected at the time of orthotopic heart transplant (OHT), (n = 7); and D) pediatric subjects with decompensated bi-ventricular failure on BiVAD support with biopsy collected at OHT (LV, RV, BiVAD), (n = 3).

**Results:**

The duration of HF was longest in OHT patients compared to BIVAD. The duration of BiVAD support was 4.3±1.9 days. Myostatin expression was significantly increased in LV-OHT compared to RV-OHT and RVOT, and was increased more than double in decompensated biventricular HF (BiVAD) compared to both OHT and RVOT. An increased myostatin/IGF-1 ratio was associated with ventricular dysfunction.

**Conclusions:**

Myostatin expression in increased in CHD, and the myostatin/IGF-1 ratio increases as ventricular function deteriorates. Future investigation is necessary to determine if restoration of the physiologic myostatin/IGF-1 ratio has therapeutic potential in HF.

## Introduction

Myostatin, a member of the TGF-β family of proteins, has been well-established as a negative regulator of skeletal muscle mass [1]. In addition, myostatin has been demonstrated to be upregulated in myocardial tissue in both small and large animal models of heart failure (HF) [2], [3], [4], where it has been proposed to play a role in the regulation of cardiac remodeling. Indeed, inhibition of myostatin in the *mdx* mouse, a model of Duchenne muscular dystrophy (DMD), accelerates the development of dilated cardiomyopathy [5]. Recently, we demonstrated that myostatin activation is increased in adult patients with advanced HF and after mechanical unloading and that myostatin latent complex is increased in the serum of adult patients in HF [6]. Other investigators have also reported that myostatin is increased in the serum [7], heart [8], and skeletal muscle [9] of adult HF patients.

To date, the regulation of myostatin in congenital heart disease (CHD) has not been described. Therefore, in this study, our goal was to quantify myostatin expression in CHD in patients with both compensated LV failure and with decompensated biventricular failure on BiVAD support. In addition, we also sought to quantify IGF-1 expression, as the relationship between myostatin and IGF-1 in the heart is poorly understood [10], [11].

## Methods

### Ethics Statement

This study met all institutional guidelines and New York State organ donation guidelines regarding use of clinical data, ethical treatment of patients adhering to Declaration of Helsinki principles, and procurement of tissue for research. This study was approved by the Institutional Review Board of Columbia University Medical Center (IRB-AAAF0646, IRB-AAAD6369, IRB-AAAF9485), the New York State Organ Donor Network, and met all institutional guidelines regarding handling of tissue specimens, HIPAA security of clinical information. Tissue from all subjects was collected at Columbia University Medical Center, while tissue from rejected organ donors was collected at local harvest sites, in accordance to New York Organ Donor Network guidelines.

Consent for this study for pediatric study subjects (IRB-AAAF0646) was waived by the Columbia University Medical Center Institutional Review Board for the following reasons: 1) all tissue collected was tissue that would otherwise have been discarded, 2) the difficulty of pre-operatively predicting which surgery would yield appropriate type and amount of specimen tissue was too high to require consent for all congenital patients undergoing surgery (our yield for tissue was ∼10/400 surgeries), and 3) the study posed no risk to subjects (to reiterate, all tissue samples were tissue that would otherwise have been discarded), and 4) all tissue and clinical data was de-identified. For adult donor hearts (IRB-AAAD6369, IRB-AAAF9485), consent for research was documented by the New York State Organ Donor Network; the Columbia University Medical Center Institutional Review Board waived consent for adult donor hearts, given that consent for research was already documented by the New York Organ Donor Network and that tissue would otherwise have been discarded. No identifying information from these patients was collected.

### Study Design

Myocardial biopsies were prospectively collected from four patient cohorts: A) adult subjects with no known cardiopulmonary disease (left ventricle, LV), (Adult Normal), (n = 5); B) pediatric subjects undergoing congenital cardiac surgery with normal RV size and function (right ventricular outflow tract, RVOT), (n = 3); C) pediatric subjects with worsening but hemodynamically stable LV failure [LV and right ventricle (LV, RV,)], with biopsy collected at time of orthotopic heart transplant (OHT), (n = 7); and D) pediatric subjects with decompensated bi-ventricular failure on BiVAD support with biopsy collected at the time of OHT (LV, RV, BiVAD), (n = 3).

### Study Subjects and Myocardial Tissue Collection

#### Normal Subjects

Subjects with no known cardiopulmonary disease whose organs were listed but were unable to be placed at the time of organ recovery for heart transplantation and who consented to tissue for research purposes by the New York Organ Donor Network were included in this study from 2004 to 2006. Organs were unable to be placed primarily due to logistical and timing constraints and matching incompatibilities - no organ was rejected from transplantation due to depressed ventricular function. At the time of organ recovery, cardioplegia with Celsior solution was administered in the standard fashion. A section (40 mg) of the left ventricle (LV) apex was immediately obtained at the conclusion of cardioplegia perfusion and flash frozen in liquid nitrogen for analysis.

#### Pediatric Patients

Children with complex CHD requiring cardiac surgery, cardiomyopathy with LV failure requiring orthotopic heart transplantation (OHT), or biventricular heart failure necessitating BiVAD support prior to OHT were enrolled in this study from 2007–2010 at the Children's Hospital of New York, Presbyterian Hospital - Columbia Campus. In patients undergoing complex CHD surgery, a sample of right ventricular outflow tract (RVOT) was obtained and flash frozen in liquid nitrogen for analysis. The Thoratec CentriMag (Pleasanton, CA) continuous flow pump was implanted in all patients. In both LV failure and BiVAD patients, myocardial samples were obtained at the time of cardiac transplantation/BiVAD explantation after cardioplegia infusion, and subsequently flash frozen in liquid nitrogen for analysis. Trans-esophageal echocardiography was performed on all patients at the time of surgery. Ventricular dysfunction and size was graded on a scale of 1–4 (1-normal, 4 abnormal) and interpreted by a pediatric cardiology echocardiographer. This grading system was used to overcome the limitations of quantitative echocardiographic measurements of RV function and to provide a tiered, discrete valuation of ventricular function solely for the purposes of graphically illustrating the relationship of myostatin/IGF-1 levels to myocardial dysfunction. None of the pediatric echocardiographic readings provide a quantitative measure of RV function, although LVEF is universally given. However, we thought it was vitally important for this study to include an analysis of RV function. Since use of a continuous variable (EF) for the RV was thus not available, we chose to quantify ventricular function according to the following system.

**LVEF:**


 Ejection Fraction    Grade
 >50                  1
 35–50                2
 20–35                3
 10–20                4


**RVEF:**


 Echo Description                                 Grade
 Normal function                                  1
 Mild dysfunction                                 2
 Mild dysfunction with mild dilation              2.5
 Moderate dysfunction with mild dilation          3
 Moderate dysfunction with moderate dilation      3.5
 Severe dysfunction, severely dilated             4


### Western Blot Analysis

Cardiac biopsies obtained for Western blotting were snap-frozen in liquid nitrogen and prepared as previously described [6]. Briefly, biopsies were weighed and homogenized in 10 uL/ug of lysis buffer for consistent protein extraction. Next, protein concentration of the extract was determined, and an equal amount of protein was loaded per sample (25 ug). Western blot was then performed for the protein of interest (i.e., myostatin, IGF-1, MEF-2) as well as for a housekeeping gene (GAPDH) to serve as a loading control, and data are reported as the ratio of, for example, myostatin/GAPDH. An internal control sample was also run on each individual gel to control for gel to gel variation. Finally, statistical analysis was performed using p<0.05 as a cutoff for significance using one-way ANOVA with post-hoc Bonferroni analysis. The following antibodies were used: myostatin (1∶500, Millipore, Temecula, CA), IGF-1 (1∶500, R&D Systems, Minneapolis, MN), MEF-2 (1∶1000, Santa Cruz Biotechnology, Santa Cruz, CA), and GAPDH (1∶4000, Santa Cruz Biotechnology, Santa Cruz, CA). All Western blots were performed in duplicate to ensure reproducibility of results.

### Statistics

Continuous variables are expressed as mean ± standard error (SE) and were compared using independent two-tailed t-testing or Wilcoxon-Mann-Whitney non-parametric testing, and one-way ANOVA with post-hoc Bonferroni analysis, when necessary. Categorical variables were compared by χ^2^. For all analyses, a *p* value of less than 0.05 was considered statistically significant. All data were analyzed utilizing SPSS 11.5 (SPSS, Chicago, IL).

## Results

### Demographics

Demographic data for normal subjects and pediatric patients from which myocardial tissue samples were obtained are summarized in Table 1. Normal adult subjects had an average age of 39.8±6.2 years, while RVOT patients were much younger than both OHT and BiVAD patients (8.2±3.0, 105.9±38.4, 89.4±27.7 months, respectively). The etiology of disease in RVOT included Ebstein's Anomaly, double outlet RV, and Tetralogy of Fallot; all BiVAD and 3 OHT patients had dilated cardiomyopathy (DCM). One RVOT patient was maintained on a single diuretic, in distinct contrast to a much sicker OHT and BiVAD population which required multiple diuretics, inotropes, and maximal vasopressors (BiVAD). One BiVAD patient was on extracorporeal membrane oxygenation prior to BiVAD implantation. Clinical and echocardiographic data are presented in Table 2. The duration of heart failure was markedly longer in OHT compared to BiVAD patients (32.3±19.9 vs. 11.7±7.0 months) (*p*<0.05). Pre-OHT/BiVAD LV function was poor in both OHT and BiVAD patients (Left ventricular ejection fraction (%): 26±7.5 and 18±4.3, respectively), but only BiVAD patients displayed severe RV dysfunction. RV function in OHT and RVOT was mildly abnormal. BiVAD patients were supported for a mean of 4.3±1.9 days prior to transplantation. All BiVAD patients were successfully bridged to transplantation.

**Table 1:** Myocardial Tissue Study Demographics.

		Adult Normal	RVOT	OHT	BiVAD
n		5	3	7	3
Age (mos)		39.8±6.2	8.2±3.0*	105.9±38.4* †	89.4±27.7* †
Weight (kg)		72.3±21.1	7.5±0.6*	31.8±9.9* †	27.6±8.7* †
Male		2	1	3	3
Etiology		n/a	Ebstein's Anomaly	DCM (3)	DCM (3)
			DORV	Myocarditis	
			TOF	Restrictive CM	
				HLHS	
				Hypoplastic TV	
Status at					
OHT/BiVAD	Inotropes	n/a	0	6†	3†
	Diuretics	n/a	1	6†	3†
	ECMO	n/a	0	0	1
PSH		n/a	Central shunt	Norwood/BDG	none
				BTS	
				TVR	

RVOT-Right Ventricular Outflow Tract, OHT-Orthotopic Heart Transplantation, BiVAD-Biventricular Ventricular Assist Device, DORV-Double Outlet Right Ventricle, TOF-Tetralogy of Fallot, DCM-Dilated Cardiomyopathy, HLHS-Hypoplastic Left Heart Syndrome, TV-Tricuspid Valve, ECMO-Extracorporeal Membrane Oxygenation, PSH-Past Surgical History, BDG-Bidirectional Glenn shunt, BTS-Blalock-Taussig Shunt, TVR-Tricuspid Valve Replacement.

*p<0.05 vs. Adult Normal,

† *p*<0.05 vs. RVOT,

**Table 2:** Clinical and Echocardiographic Data.

	Adult Normal	RVOT	OHT	BiVAD
Pre-OHT/LVAD	n/a	n/a	32.3±19.9	11.7±7.0*
Duration of HF (mos)				
Pre-OHT/BiVAD LV	60±4.0	53±1.7	26±7.5†	18±4.3* †
Ejection Fraction (%)				
Pre-OHT/BiVAD LV	1.0±0.0	1.0±0.0	3.0±0.4†	4.0±0.0* †
Dysfunction^a^				
Pre-OHT/BiVAD RV	1.0±0.0	1.7±0.7‡	1.5±0.2‡	4.0±0.0* †
Dysfunction^a^				
Duration of VAD	n/a	n/a	n/a	4.3±1.9
support (days)				
LVEDD (cm)	n/a	2.9^b^	4.2±1.0	5.9±1.5
IVSD (cm)	n/a	0.47^b^	0.69±0.06	0.63±0.07
PWT (cm)	n/a	0.33^b^	0.67±0.07	0.65±0.15

BiVAD-Biventricular Ventricular Assist Device, HF-Heart Failure, LV-Left Ventricular, RV-Right Ventricular, OHT-Orthotopic Heart Transplantation, LVEDD-Left Ventricular End Diastolic Diameter, IVSD-Interventricular Septal Dimension, PWT-Posterior Wall Thickness.

*p<0.05 vs. OHT,

† *p*<0.05 vs. Adult Normal, RVOT,

‡ *p*<0.05 vs Adult Normal.

a Details of this grading system can be found in the Methods section.

b Two RVOT patients–one with DORV and one with TOF–both had very large muscular VSD's, and echocardiography was limited in these patients. As a result, these values represent data from one RVOT patient.

### Myostatin Expression

**Figure 1:**
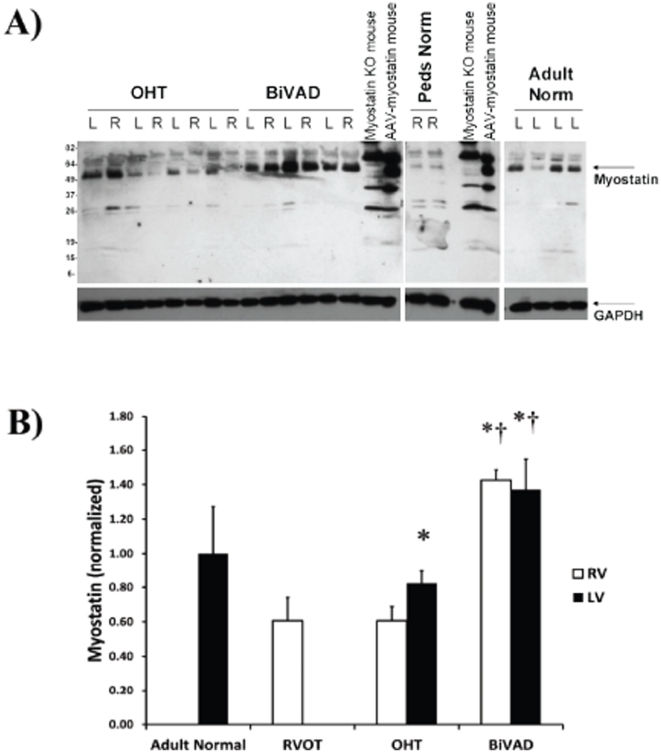
Myostatin Expression. A.) Representative Western blot for the myostatin peptide (∼50 kDa) in Adult Normal (n = 5), pediatric right ventricular outflow tract (RVOT) (n = 3), pediatric orthotopic heart transplant (OHT) (n = 7 paired LV and RV), and pediatric biventricular assist device (BiVAD) myocardial tissue samples (n = 3 paired LV and RV). B.) No significant differences were found between Adult Normal and RVOT, while LV-OHT were significantly higher than RV-OHT samples (*p*<0.05). BiVAD support increased myostatin more than twice the levels observed in OHT or RVOT in both LV and RV samples (all *p*<0.05). (*p<0.05 vs. RVOT, RV-OHT, † *p*<0.05 vs. LV-OHT).

Myostatin ran at approximately 50 kDa, an apparent molecular weight that was somewhat higher than predicted, most likely due to glycosylation (Figure 1A) [12]. Antibody specificity was confirmed by running samples from a myostatin knock-out mouse heart and myostatin overexpressing mouse heart along with the human samples. No significant difference was observed when comparing Adult Normal and pediatric RVOT controls (Figure 1B). Myostatin expression was increased in LV-OHT vs. both RV-OHT and RVOT control (Figure 1B) (p<0.05). In addition, myostatin expression was increased over two-fold in decompensated HF (BiVAD) vs. both OHT and RVOT control (Figure 1B) (p<0.05).

### IGF-1 Expression

**Figure 2:**
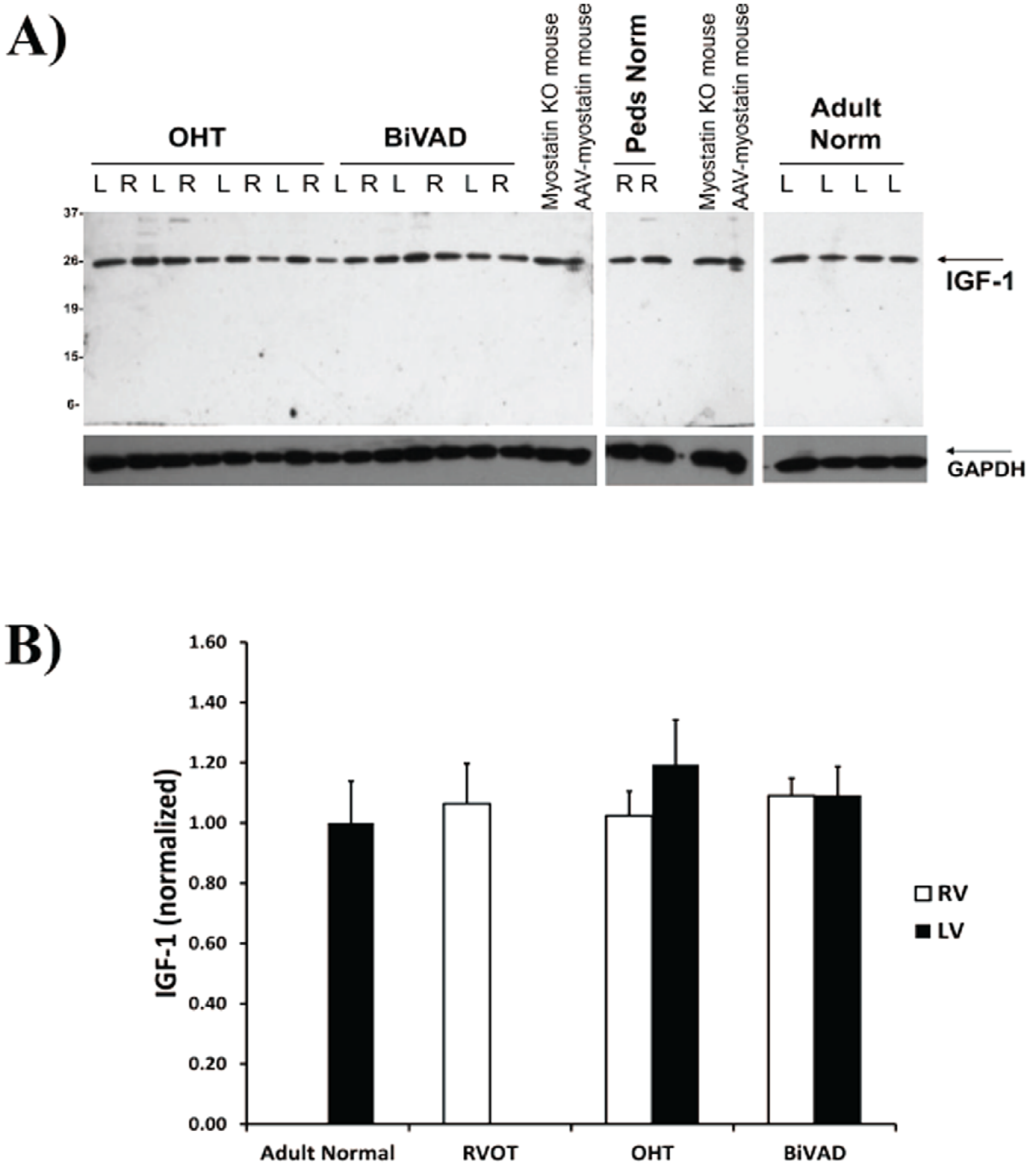
IGF-1 Expression. A.) Representative Western blot for IGF-1 expression in Adult Normal (n = 5), pediatric right ventricular outflow tract (RVOT) (n = 3), pediatric orthotopic heart transplant (OHT) (n = 7 paired LV and RV), and pediatric biventricular assist device (BiVAD) myocardial tissue samples (n = 3 paired LV and RV). B.) No significant difference in IGF-1 expression was observed in any cohort.

**Figure 3:**
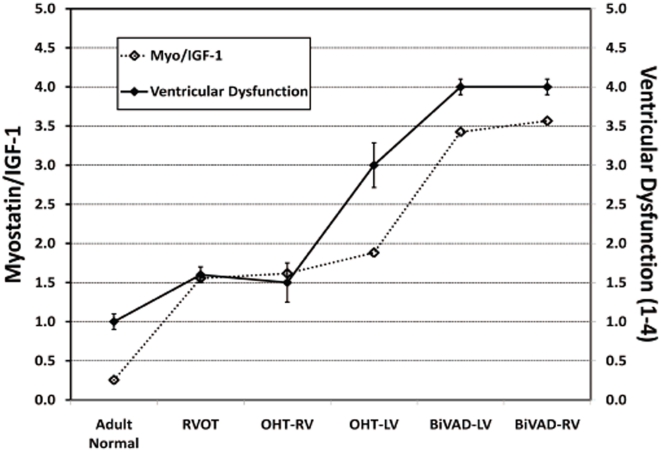
Myostatin to IGF-1 Ratio. The relationship of myostatin/IGF-1 (left axis) and ventricular dysfunction (right axis) has been plotted for Adult Normal (n = 5), pediatric right ventricular outflow tract (RVOT) (n = 3), pediatric orthotopic heart transplant (OHT) (n = 7 paired LV and RV), and pediatric biventricular assist device (BiVAD) myocardial tissue samples (n = 3 paired LV and RV). A strong association between increased myostatin/IGF-1 ratios and worsening ventricular dysfunction exists: Adult Normal samples had low myostatin/IGF-1 levels, while BiVAD samples displayed high myostatin/IGF-1 levels.

A link between myostatin and IGF-1 in the failing heart has been suggested in the literature [10], [11]. The IGF-1 band ran at approximately 25 kDa, which is the size reported by other investigators using the same antibody (Figure 2A) [13]. No significant difference was noted when comparing IGF-1 expression in pediatric OHT and pediatric BiVAD vs. RVOT or Adult Normal (Figure 2B). Next, we calculated the ratio of myostatin to IGF-1 and plotted this value as a function of ventricular dysfunction. We found that the myostatin/IGF-1 ratio was significantly associated with ventricular function: as the myostation/IGF-1 ratio increased, ventricular function deteriorated (R^2^ = 0.91, R^2^ = 0.91, respectively) (Figure 3).

### MEF-2 Expression

**Figure 4:**
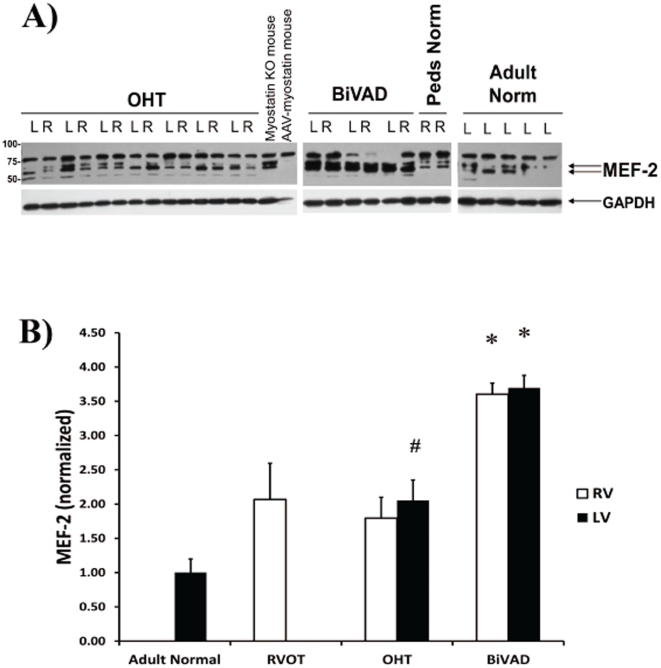
MEF-2 Expression. A.) Representative Western blot for MEF-2, a transcriptional factor for myostatin, in Adult Normal (n = 5), pediatric right ventricular outflow tract (RVOT) (n = 3), pediatric orthotopic heart transplant (OHT) (n = 7 paired LV and RV), and pediatric biventricular assist device (BiVAD) myocardial tissue samples (n = 3 paired LV and RV). B.) MEF-2 was significantly increased in LV-OHT compared to Adult Normal control (#*p*<0.05). In addition, BiVAD support increased MEF-2 significantly over all patient groups in both RV and LV samples (*p<0.05 vs. Adult Normal, RVOT, RV-OHT, LV-OHT).

MEF-2 is a transcription factor that has been implicated in the regulation of myostatin expression [10], [11]. MEF-2 ran as a doublet at approximately 65 kDa (Figure 4A). MEF-2 expression was significantly higher in LV-OHT compared to Adult Normal (Figure 4B), and MEF-2 expression was significantly higher in pediatric BiVAD vs. pediatric OHT, RVOT, and Adult Normal (Figure 4B). In addition, expression of MEF-2 was upregulated in myostatin knock-out mice and suppressed in mice overexpressing myostatin (Figure 4A).

## Discussion

To our knowledge, this is the first study investigating myostatin regulation in the pediatric heart, and our primary findings in this small cohort of patients are (i) myostatin expression is increased in compensated LV HF (LV-OHT) compared to paired normal RV control (RV-OHT) and unpaired normal RV control (RVOT), (ii) myostatin expression is increased in decompensated biventricular HF after mechanical unloading (BiVAD) compared to both compensated HF (OHT) and control (RVOT), (iii) the myostatin/IGF-1 ratio increases in correlation with the degree of ventricular dysfunction, and (iv) MEF-2 expression is increased in compensated LV HF (LV-OHT) vs. normal LV (Adult Normal) and further increased in decompensated biventricular failure after mechanical unloading (BiVAD).

The role of myostatin in HF has not yet been definitively determined [14]. Accumulating evidence suggests that myostatin may be a critical regulator of cardiac remodeling. In animals, it has been observed that myostatin is upregulated following infarct in sheep and rats [2], [15], volume overload in rats [3], and pressure overload in mice [4]. In addition, inhibition of myostatin in the *mdx* model of DMD accelerates the onset of systolic dysfunction and LV chamber dilatation [5]. In humans, myostatin has been reported to be increased in the serum [7], heart [8], and skeletal muscle [9] of adult HF patients, although one study did report decreased serum myostatin in HF patients [16]. In addition, we have previously reported that myostatin activation is increased in adult HF of both ischemic and non-ischemic etiologies [6], and now we have determined that myostatin expression is increased in HF secondary to CHD. Additional studies will need to be performed in which myostatin is both inhibited and overexpressed in an animal model to determine its exact role in HF as well as the therapeutic potential of manipulation of myostatin expression in this setting.

It is interesting to note the much larger relative increase in myostatin after BiVAD support versus LVAD support [6]. The loss of feedback inhibition after mechanical unloading appears more pronounced with biventricular failure–whether different inhibitory pathways are activated/de-activated or other stress pathways are involved remains under investigation. This increase in myostatin following mechanical unloading may have been expected given the fact that exercise training (i.e. loading) results in decreased skeletal muscle myostatin in healthy humans and in several pathological states [17], including HF [9] and insulin resistance [18], as well as in decreased skeletal and cardiac muscle myostatin expression in a rat model of HF [15]. It is possible that myostatin may mediate cellular atrophy during periods of mechanical unloading and that myostatin levels must decrease to allow physiological hypertrophy during exercise training. Additonal experiments in animal models will need to be performed to test these hypotheses.

We also examined the relationship between myostatin and IGF-1 in this study and found that the ratio of myostatin to IGF-1 increased as ventricular function decreased. A link between myostatin and IGF-1 in the heart has been previously suggested [10], [11]. Shyu *et al* found that myostatin is increased following cardiomyocyte stretch in culture and that this increase in expression is dependent upon IGF-1 secretion and subsequent signaling through the p38 pathway [11]. Based on these data, a model has been proposed whereby IGF-1 signals preferentially via Akt under physiologic conditions to promote physiologic hypertrophy and/or cell survival. There is also likely baseline signaling through p38, which leads to increased myostatin expression via the MEF-2 transcription factor, thus exerting negative feedback on the Akt growth pathway via PTEN [11], [19], [20]. However, under pathologic conditions such as pressure and/or volume overload in the failing heart, an unknown trigger may occur, which causes IGF-1 to signal predominantly through the p38/MEF-2 pathway and which results in increased expression of myostatin out of proportion to IGF-1. The stimulus for this trigger may be pathologic stretch in HF, as suggested by Shyu *et al* [11]. If this is the case, we believe that an increased myostatin to IGF-1 ratio may be a clinical marker of worsening HF. Indeed, we observed increased MEF-2 in conditions where we also observed increased myostatin, which supports the proposed pathway. Future effort will be directed towards examining p38 and Akt signaling in these patients.

It is unclear at this point whether the increased myostatin to IGF-1 ratio causes or contributes to ventricular dysfunction in HF. It is known that decreased activity of the growth hormone (GH)/IGF-1 axis (low IGF-1 syndrome) is predictive of poorer outcomes and higher mortality in HF [21], and that GH replacement, which increases IGF-1 expression, can lead to increased peak oxygen uptake and exercise duration as well as higher quality of life [22]. This therapy may function by restoring the physiological myostatin/IGF-1 ratio and overcoming pathologic repression of IGF-1 by myostatin. Our findings represent a small sample cohort, and additional experiments in which expression of both myostatin and IGF-1 is manipulated will need to be performed in an animal model of HF to delineate the significance of the myostatin/IGF-1 ratio in HF further.

In summary, we have demonstrated for the first time that myostatin expression is increased in pediatric compensated LV failure and further increases in decompensated biventricular failure after mechanical support. In addition, we observed that the myostatin/IGF-1 ratio may increase as ventricular function decreases. Future investigation is needed to determine the effects of myostatin inhibition and overexpression in HF.
